# Mesenchymal Stromal Cells for Graft Versus Host Disease: Mechanism-Based Biomarkers

**DOI:** 10.3389/fimmu.2020.01338

**Published:** 2020-06-25

**Authors:** Tik Shing Cheung, Giuliana Minani Bertolino, Chiara Giacomini, Martin Bornhäuser, Francesco Dazzi, Antonio Galleu

**Affiliations:** ^1^School of Cancer and Pharmacological Sciences and KHP Cancer Research UK Centre, King's College London, London, United Kingdom; ^2^University Hospital Carl Gustav Carus, TU Dresden, Dresden, Germany

**Keywords:** mesenchymal stromal cell, graft versus host disease, biomarker, apoptosis, efferocytosis, extracellular vesicles

## Abstract

The immunosuppressive activity of mesenchymal stromal cells (MSCs) in graft versus host disease (GvHD) is well-documented, but their therapeutic benefit is rather unpredictable. Prospective randomized clinical trials remain the only means to address MSC clinical efficacy. However, the imperfect understanding of MSC biological mechanisms has undermined patients' stratification and the successful design of clinical studies. Furthermore, although MSC efficacy seems to be dependent on patient-associated factors, the role of patients' signature to predict and/or monitor clinical outcomes remains poorly elucidated. The analysis of GvHD patient serum has identified a set of molecules that are associated with high mortality. However, despite their importance in defining GvHD severity, their role in predicting or monitoring response to MSCs has not been confirmed. A new perspective on the use of MSCs for GvHD has been prompted by the recent findings that MSCs are actively induced to undergo apoptosis by recipient cytotoxic cells and that this process is essential to initiate MSC-induced immunosuppression. This discovery has not only reconciled the conundrum between MSC efficacy and their lack of engraftment, but also highlighted the determinant role of the patient in promoting and delivering MSC immunosuppression. In this review we will revisit the extensive use of MSCs for the treatment of GvHD and will elaborate on the need that future clinical trials must depend on mechanistic approaches that facilitate the development of robust and consistent assays to stratify patients and monitor clinical outcomes.

## A Brief History of MSCs

Mesenchymal stromal cells (MSCs) are typically described as a highly heterogeneous population of stem and progenitor cells selected and expanded *in vitro* as unfractionated fibroblastic-like and plastic-adherent cells (1). This population was first described in the early 1970s by Friedenstein and colleagues who isolated from the bone marrow (BM) of guinea-pigs and mice a group of fibroblastoid cells able to differentiate into adipocytes, chondrocytes and osteocytes and to reconstitute the microenvironment for the culture of hematopoietic stem cells (HSC) (2, 3). These cells were later identified in human tissues (4) and referred to as mesenchymal stromal cells (MSCs) (5).

Since their first description, cells with analogous characteristics have been successfully isolated and expanded from many other tissues (6), such as placenta (7), umbilical cord (UC) (8), adipose tissue (AT) (9), and dental pulp (10). MSC identification relies on the combined expression of CD73, CD90, CD105, CD71, CD44, CD106, and the lack of hematopoietic and endothelial markers (CD34, CD45, CD11b, CD14, and CD31) (11). The definition of MSCs features a substantial overlap with the traditional concept of other more mature stromal cells, such as fibroblasts (12–14), making it plausible to consider that these are equivalent or related cell types.

MSC heterogeneity has been described within the same species (15), tissue preparations (16, 17) and even on same donor isolations. As observed by Mets and Verdonk, during MSC sub-cultivation, younger passages were characterized by higher rates of plasticity and proliferation compared with older passages (18). Yang et al. (19) also described gradual loss of the typical fibroblast-like spindle shape, decreased expression of the adhesion molecule CD146 and genetic instability in human MSCs under increasing *in-vitro* passages. Despite their heterogeneity, MSCs have been largely employed in experimental cell-based therapies for treating human diseases. Historically, the lack of the expression of major histocompatibility complex (MHC) class II and co-stimulatory molecules (CD40, CD40L, CD80, and CD86), associated with low levels of MHC class I on MSC surface (20, 21), initially introduced the idea of a population of “immune-privileged” cells which could be widely used beyond MHC-compatibility restrictions (22), and this consideration greatly ignited the enthusiasm around MSCs as therapeutic tools.

The possibility that MSCs might be devised as therapeutically effective cellular products mainly derived from studies describing MSC ability to improve tissue healing and regeneration (23, 24) and to alter host immune responses by suppressing inflammation (22, 25–28). It has not been fully elucidated how healing and immune suppressive MSC properties are intertwined. However, they are not mutually exclusive or completely independent as tissue regeneration requires resolution of injury-associated inflammation. In this review, the multipotency of MSCs will not be further discussed [reviewed in Bianco et al., (29) and Caplan (30)]. Conversely, immunosuppression mediated by MSCs will be extensively examined.

It is widely accepted that MSC immunosuppressive properties are not constitutive. Instead, their immune regulation depends on a process of “licensing” which needs to be acquired in an inflammatory microenvironment. This concept finds support not only *in vitro* but also in preclinical models of graft versus host disease (GvHD), whereby MSC therapeutic activity could be obtained only when MSCs were infused in the presence of a specific inflammatory milieu (31). Accordingly, MSCs were effective in reducing GvHD signs only when multiple infusions were administered after transplant but not when one single dose was co-infused with HSC transplantation (HSCT) (32). These experimental observations have been strongly supported by a meta-analysis recently performed by Wang and collaborators (33). In this work, the authors included 6 randomized clinical trials enrolling 365 patients. MSCs were infused at different time points from HSCT (within 24 h, at a median time of 28 days, or with multiple infusions at different time points). The analysis showed that infusion of MSCs significantly reduced the incidence of chronic GvHD (cGvHD) and there was a trend of longer overall survival in MSC-treated patients (33). Importantly, the meta-analysis on different sub-groups demonstrated that these favorable outcomes were significantly associated with late MSC administrations, thus supporting a more effective role of MSCs when licensed by a specific microenvironment developed after HSCT.

Once licensed, MSCs are able to mediate potent immunoregulatory effects through diverse modes of action on a variety of cell types, involving both the adaptive and innate immunity. The immunomodulatory repertoire induced by primed MSCs includes factors such as indoleamine 2,3-dioxygenase (IDO) (34–36), prostaglandin E2 (PGE2) (28, 34), heme oxygenase-1 (HO-1) (37), transforming growth factor beta (TGF-β), IL-10 (38), human leukocyte antigen-G5(HLA-G5) (39), leukemia inhibitory factor (LIF) (40), and galectin 1, 3, and 9 (41–43). IDO, PGE2 and HO-1 can directly induce metabolic reprograming on activated T lymphocytes, reducing their proliferation rates, cytokine production and cytotoxic activity (28, 34, 37, 44). Likewise, MSCs suppress the proliferation and modulate the cytokine production of activated natural killer (NK) cells (45, 46) through the action of IDO and PGE2 (28, 34, 36, 47). Furthermore, MSCs can inhibit B-cell activation, proliferation and IgG secretion both *in vitro* and *in vivo* (44, 47) in a soluble-factor dependent manner (48). In addition, MSCs can dampen the activation of effector immune cells via cell-contact interactions through the association of the programmed death 1 and its ligand (PD-1/PDL1) (49, 50) and HLA-G1 (51).

Reprogramming of the host immune cells is another mean of MSC immunomodulation, especially *in vivo*. MSCs can recruit monocytes to the site of inflammation by the secretion of chemokine ligand 2 (CCL2) (52). In a heart injury model, MSCs reduced the number of pro-inflammatory monocytes, while increased those with anti-inflammatory phenotype (53). Moreover, in the presence of colony stimulating factor 1 (CSF-1/M-CSF), MSCs can promote monocyte differentiation into macrophages with upregulated expression of CD206, IL-10, and TGF-β and improve phagocytic efficiency, which suggests the characteristics of M2 macrophage differentiation (54). Likewise, bone marrow progenitors are induced to differentiate into a population of CD11b+ myeloid cells with potent suppressive activity in the presence of MSCs. Such differentiation is mediated by nitric oxide synthase-2 and these MSC-educated CD11b+ cells accelerate hematopoietic reconstitution in bone marrow transplant recipients (55).

MSCs can also inhibit monocyte differentiation into dendritic cells (DCs) and skew them into a tolerogenic profile via reducing their expression of HLA-DR, CD1a, CD80, and CD83 as well as down-regulating their IL12 secretion (56–59). Moreover, effects of MSCs on regulatory T cells (Treg) expansion have also been documented in many inflammatory conditions (60–62). MSCs induce differentiation of functional Treg through TGF-β, HLA-G5, IDO, and PGE2 (39, 59, 63). Remarkably, MSCs can further favor Treg expansion *in vitro* indirectly by inhibiting DC maturation, CD8 T cells, and NK cells expansion (59, 64).

The up-stream mechanism of MSC licensing has been a puzzle for decades in MSC research. Pro-inflammatory cytokines such as IFN-γ, TNF-α, IL-1α, or IL-1β have been extensively reported on MSC activation *in vitro*. These molecules are predominately secreted by activated monocytes or T cells, and subsequently trigger the production of immunosuppressive molecules in MSCs (35, 36, 65, 66). Blockage on either these soluble factors or cell-contact pathways successfully undermined MSC immunomodulatory effects. Yet, generally none of these molecules taken alone is sufficient to account for MSC suppressive function which in fact seems to be the result of a synergistic combination of more factors. Therefore, how these molecules are intertwined *in vivo* and the degree of redundancy of their effects on MSC licensing warrants further investigation.

## Current Challenges Associated With the Use of MSCs For the Treatment of GvHD

### MSC Use as a Therapeutic Tool

**Figure 1:**
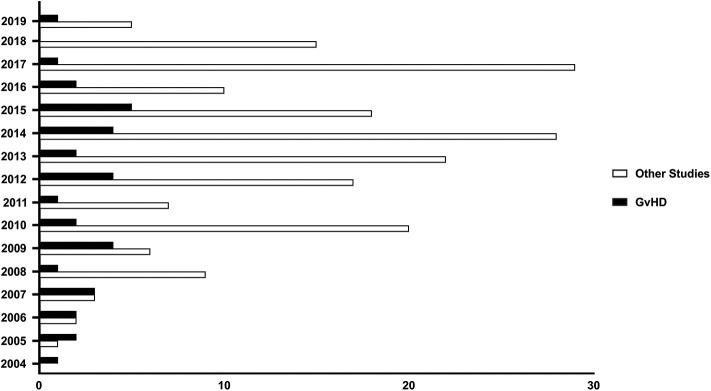
MSCs as therapeutic agents in immune-mediated diseases. Number of Clinical trials registered at the U.S. NIH database registry (ClinicalTrials.gov) plotted according to the year of registration. Search was performed in August 2019 and included all studies whereby MSCs (Mesenchymal Stromal/Stem Cells) were used as drug for the treatment of GvHD (black bars), or other immune-mediated diseases (white bars) such as Chron's Disease, Cystic Fibrosis, Diabetes Mellitus, engraftment of HSCT, inflammatory lung diseases (including asthma and Chronic Obstructive Pulmonary Disease), Multiple Sclerosis, neuromyelitis, Retinitis Pigmentosa, Rheumatoid Arthritis, Rheumatic arthritis, Sjogren Syndrome, solid allograft rejection, Systemic Lupus Erythematosus, Systemic Sclerosis, Ulcerative Colitis.

The characteristics described in the previous paragraph elicited the interest in MSCs, considered as promising therapeutic tools to control aberrant inflammatory responses. As shown in Figure 1, consultation of the public registry of clinical trials at the U.S. National Institute of Health database (at *ClinicalTrials.gov*) shows a continuous increase of the number of new studies involving MSCs for the treatment/prophylaxes of immune-mediated diseases which were registered between 2004 and 2010, with at least 6 new registrations thereafter.

The focus of the use of MSCs as cell-therapy products has been mainly focused on two aspects: (1) the use of MSCs to exert peripheral tolerance in contexts whereby this tolerance was altered after the use of MSCs (i.e., usefulness of MSCs as prophylaxes), and (2) the use of MSCs when an inflammatory or autoimmune response was already established before MSC infusion (i.e., MSC use as specific therapy to restore peripheral tolerance). Aim of this review is to focus on the use of MSCs after HSCT and in GvHD patients.

### MSCs for the Treatment of GvHD

GvHD is a life-threatening complication of allogeneic HSCT, and currently represents one of the major factors limiting the success of this potentially curative option for hematological malignancies (67, 68). GvHD has been classified into acute (aGvHD) and cGvHD (69, 70). Currently, there is no standardized treatment for patients with aGvHD who do not respond to steroids, and their prognosis is still very poor, with overall survival inferior to 20% at 2 years (71). The interest in MSCs for the treatment of aGvHD has sparked remarkably since the very encouraging results published in 2008 by the European Group for Blood and Marrow Transplantation Developmental Committee (72), with 30 out of 55 patients with steroid resistant aGvHD showing complete response to MSCs. Importantly, these responding patients had 55% overall survival at 2 years. To date (last analysis in September 2019), at least one new clinical trial involving the use of MSCs to mitigate GvHD has been registered every year at ClinicalTrial.gov with a peak of 5 different studies started in 2015 (Figure 1). A systematic search of the published manuscripts in peer-reviewed journals has identified 14 studies (72–85), both retrospective and interventional, with more than 30 patients enrolled. In these studies, all aGvHD patients were steroid-resistant. Only one study used MSCs as first line treatment in association with steroids (76). Both pediatric and adult patients were treated with age ranging from 2 months to 72 years. It is not possible to directly compare these studies in terms of efficacy due to the heterogeneity of the patients enrolled. However, results seem to be very encouraging. Indeed, as summarized in Table 1, overall response rates ranged from 47 to 93% even though patients were mostly resistant to multiple lines of treatments. Notably, the use of MHC-matched, haploidentical or third-party MSC donors does not have any impact on patient outcomes (72–85).

**Table 1:** Clinical studies with MSCs used in aGvHD.

	**Patients**		**MSC infusion**		**Outcome**		
**Publication**	***N***	**Median age (range)**	**Dose (×10^**6**^/Kg)**	**Median (range)**	**CR (%)**	**PR (%)**	**NR (%)**
LeBlanc et al. (72)	55	22 (0.5–64)	0.40–9.00	2 (1–5)	54	16	29
Ball et al. (73)	37	7 (0.7–18)	0.90–3.00	2 (1–13)	65	22	13
Kurtzberg et al. (74)	75	8 (0.2–17)	2.00	NRe (8–12)	NRe	NRe	NRe
TeBoom et al. (75)	48	44.9 (1.3–68.9)	1.80 (0.90–2.50)	3 (1–4)	25	50	25
Kebriaei et al. (76)	31	52 (34–67)	2.00–8.00	2 (2)	77	16	7
Erbey et al. (77)	33	7 (3–18)	0.50–2.80	2 (1–4)	54	21	25
Servais et al. (78)	33	58 (5–69)	NRe (1.00–4.00)	1 (1–2)	22	41	37
vonDalowski et al. (79)	58	55 (19–71)	0.99 (0.45–2.08)	2 (1–6)	9	38	53
Dotoli et al. (80)	46	28 (1–72)	6.81 (0.98–29.78)^**^	3 (1–7)	7	43	50
Bader et al. (81)	69	8.2 (6 mo-18) 45.5 (18.9–65.5)	NRe (1.00–2.00)	NRe (1–4)	32	51	14^*^
Introna et al. (82)	37	27.8 (1–65)	NRe (0.80–3.10)	NRe (2–11)	30	43	27
Fernandez-Mazqueta et al. (83)	33	46 (18–61)	1.06 (0.66–1.76)	1.06 (0.66–1.76)	34	50	16
Resnick et al. (84)	50	19 (1–69)	1.00 (0.3–3.10)	NRe (1–4)	34	32	34
Galleu et al. (85)	60	40 (4 mo-68)	2.60 (0.60–15.60)	1 (1–4)	2	52	46

*CR, Complete Response; NRe, Not reported; NR, No Response; PR, Partial Response*;

* *3%, no data available at day 28*;

** *cumulative dose*.

MSCs could successfully be expanded from disparate tissues, spanning from BM (72–82, 84), UC (86, 87), AT (88, 89), or placenta (90). BM has been the first MSC source ever described and the most frequently deployed thus far. However, the origin of MSCs does not seem to affect their anti-proliferative and immunological properties *in vitro* (91). Furthermore, despite the small number of patients treated with UC (86, 87), AT (88, 89) or placenta (90), similar response rates were reported when compared to the outcomes obtained when BM-MSCs were used (Table 1), thus supporting the role of these sources as valid alternatives for clinical-grade MSC production. In fact, UC and AT may be considered as more “affordable” alternative sources in terms of manufacturing logistics and costs compared to BM. Obtaining MSCs from UC or AT has important advantages. First, the invasive BM harvest procedure, associated with (minimal) risk for donors, can be spared. Secondly, both UC- and AT-MSCs can be obtained from tissues which are currently otherwise discarded and also from samples previously frozen before isolation (this at least it has been described in UC-MSCs) (92, 93). Third, they have higher proliferative capacity and longer life-span *in vitro* with higher cells yielded per expansion (94, 95).

MSC therapeutic activity has been tested also in cGvHD, albeit the experience in this setting is more limited than in aGvHD. Most studies reported the treatment of only few patients, and they should be considered as case reports. Results were in fact variable, with overall responses ranging from 0% (82, 96) to more than 50% of the patients treated (97–100). More promising are the results obtained from larger groups of patients and reported in three different studies. In two of these studies, a total of 57 steroid-refractory cGvHD were treated with 1–5 infusions of BM-MSCs. The median time to response varied between 3 and 24 months after the first MSC infusion (101, 102). Notably, 26% of the patients treated could wean immunosuppressive therapy until complete discontinuation in one of the studies (102). Recently, 14 patients with moderate to severe cGvHD were prospectively treated with one infusion of AT-MSCs as first-line treatment in association with steroids and cyclosporine (103). In total, 13 patients could be evaluable, since 1 patient withdrew participation consent. Ten patients achieved a response at 56 weeks [8 complete response [CR] and 2 partial response [PR]], all stopped steroids and were alive at the end of the study. Conversely, of the 3 non-responding patients, none was alive and the cause of death was progressive cGvHD (103).

### MSCs for the Improvement of HSCT and as Prophylaxis of GvHD

MSCs have been demonstrated to enhance haematopoietic engraftment and hematological recovery after both autologous (104) and allogenic (105–107) HSCT when administered at the time of transplant. This property may become crucial in situations in which, due to damage of the BM niche after conditioning regimens for HSCT, haematopoietic recovery may be severely delayed. Koc et al. (104) were the first to report improvement of haematopoietic engraftment when autologous BM-MSCs were co-transplanted with HSCT. These findings, along with positive results from preclinical models whereby MSCs were able to delay the onset of GvHD (108, 109), prompted investigators to assess whether MSCs could be used for the improvement of HSCT engraftment and prophylactically to decrease the frequency of GvHD when co-administered with the transplant. The ability of MSCs of improving HSCT engraftment, or preventing graft failure, seemed to be confirmed in some studies (106, 107, 110–113). However, absence of any improvement has also been reported (114, 115). Recently, a comprehensive meta-analysis carried out by Kallekleiv et al. (116) determined the potential benefits of MSCs when co-administered with allogenic HSCT within a range of 24 h (before or after the transplant). The study included a total of 309 patients enrolled in 9 controlled trials performed until May 2015, thereof 3 randomized and 6 non-randomized studies. The analysis suggests that MSCs do not have any beneficial effects in terms of facilitation of engraftment or either aGvHD nor cGvHD prevention (116). Important limitation of this meta-analysis relates to the small sample sizes of the studies included and their weak designs, thus results should be interpreted with caution.

Taken together, these data suggest that, while MSC use is safe, the efficacy of this treatment as tool to promote HSCT engraftment or GvHD prophylaxis should not be routinely supported. Factors which may play a role in influencing the activity of MSCs include the concomitant therapy, the underlying disease or the conditioning regimen. By modifying the inflammatory milieu of the patient, these components may affect the MSC “licensing” and hamper their immunomodulatory capacity to reset the haematopoietic niche.

### MSC Biomarkers for GvHD: An Unmet Need

In the previous paragraph, we have reported the very encouraging results when MSCs are used for the treatment of aGvHD. However, the only randomized phase III trials, sponsored by Osiris Therapeutics (NCT00562497 and NCT00366145) and making use of commercially available MSCs (Prochymal), missed their endpoints and failed to demonstrate efficacy of MSCs. Nonetheless, this failure was only announced by press-release and results were never published in peer-reviewed manuscripts. To make them more difficult to interpret, the publicly available results (published in abstract forms only) did demonstrate the efficacy of MSC treatment in specific sub-categories of patients with improvements in response rates in pediatric patients (117) or patients with gut or liver GvHD (118).

These results and the contrast with the outcomes reported in most phase II studies raised many questions on the possible causes of this failure (119). Furthermore, it drove to question the very same utility of MSCs as part of the available treatments of GvHD, as highlighted by the recent clinical commissioning policy on GvHD treatments published by NHS England, which concluded that there was not enough evidence for supporting the use of MSCs in GvHD patients (120). It is conceivable that to definitely and robustly assess the role of MSCs in GvHD therapeutic armamentarium, we need prospective phase III trials whose design needs to be guided by potency assays or biomarkers able to effectively stratify patients and predict clinical responses.

A biomarker (or biological marker) is a parameter that can be objectively measured or evaluated to indicate a biological process, pathogenic processes, or pharmacologic responses to a therapeutic intervention (121). In the regard of MSC therapy in aGvHD, ideal MSC biomarkers can be served as a prognostic tool to (1) forecast the clinical outcome, or (2) predict the clinical response, or (3) monitor the efficacy of MSC therapy among a variety of aGvHD patients.

There have been two major approaches to predict or monitor the therapeutic effects of MSC in GvHD. The first approach has been to apply a panel of GvHD biomarkers, which are molecules related to the tissue damage during the pathogenesis of aGvHD (122). They were first identified to provide diagnostic and prognostic information on GvHD independently of the clinical symptoms (122). The initial panel included the plasma level of interleukin 2 receptor subunit α (IL-2Rα), tumor necrosis factor receptor 1 (TNFR1), interleukin-8 (IL-8), and hepatocyte growth factor (HGF). Subsequently, the same research group included two organ–specific biomarkers, which are regenerating islet-derived 3α (Reg3α) (123) and elafin (124), specific for gastrointestinal and skin GvHD, respectively. Several studies in aGvHD have reported the change of these biomarker after the MSC infusion and their correlation with the MSC responses. For instance, Dander et al. (125) found a decrease of plasma TNFRI, IL-2Rα, and elafin in those patients who responded to MSCs but not in the non-responders. At the same year, von Bahr et al. (126) reported similar decline of serum IL-2Rα in GvHD patients after MSC infusion, although they did not compare the change of IL-2Rα between responders and non-responders. Later, Yin et al. (127) found a fall of Reg3α and cytokeratin fragment 18 (CK18), another tissue damage biomarker in liver and intestinal GvHD, in MSC responders (128). However, discrepancies have also been reported. In contrast to Yin et al. (129) another study indicated that Reg3α and IL-2Rα were not correlated with the response to MSCs in aGvHD patients. Furthermore, in a phase II study, there was no correlation of any GvHD biomarkers with the clinical response following MSC treatment in aGvHD patients (75), raising questions on the reliability of these GvHD biomarkers in monitoring MSC efficacy.

The second approach has been to monitor some effector molecules or cellular pathways reported as mediators of MSC immunosuppression *in vitro* and in pre-clinical studies. Dander et al. (125) reported an increase in the proportion of Treg compared to Th1 and Th17 cells after MSC treatment in the responders, while opposite results were found in the non-responders. However, another study did not find any increase of the Treg population in both responders and non-responders. Moreover, both the numbers and functions of CD4 and CD8 T cells also remained unchanged. The only significant difference between responders and non-responders was the proportion of immature DCs which was increased among the responders following MSC infusion (75). Similar ambiguity was also noticed in the effector molecules which have been described in MSC immunosuppression *in vitro*. For instance, no changes of the serum level of IL-6 or HLA-G was detected in patients after receiving MSCs regardless of the clinical response (126). Importantly, the same study also found that MSC immunosuppressive potency, measured as anti-proliferative activity against T cells after stimulation *in vitro*, did not correlate with MSC clinical efficacy *in vivo*. This observation indicates that the role of this *in vitro* potency assay was not optimal to predict the clinical efficacy of MSCs in aGvHD patients (126).

Albeit not strikingly biomarkers by definition, there have been several associative factors which can seemingly influence MSC responses among aGvHD patients. Dosage of MSC infusion, age of MSC recipients and organ involvement have been reported as affecting responses in aGvHD patients (72, 77, 78, 82, 84, 85, 130). Briefly, patients who received higher MSC doses (78, 85), younger patients (72, 82, 84, 85), or patients with gut or/and skin involvement seem to achieve a better response to MSCs (77, 85, 130). However, these results have not been confirmed in other studies (73, 79, 131), and should be considered with caution. These discrepancies highlight the weak reliability of these associative factors when used as predictors of response. Furthermore, the biology underlying these clinical observations is still unknown. Nevertheless, they unquestionably stress the importance of the patient as crucial player in the response.

### The Paradox of MSC Immunosuppression Models (*in vitro* vs. *in vivo*)

The unsatisfactory ability to predict or monitor clinical responses to MSCs by the panel of molecules described in the previous paragraph can be attributed to two main reasons. First, the proposed GvHD biomarkers (e.g., Reg3α and Elafin) (75, 125, 128, 132) appear to be reliable sensors of the severity of the disease and tissue damage but they lack any mechanistic rationale regarding the immunosuppressive activity of MSCs and their licensing. It is then arguable that their impact on MSC clinical response may be minimal. However, if variations of their values among responders and non-responders are consistently found in larger studies, they will acquire a more defined role in the monitoring of the GvHD after MSC treatment.

Second, the immunosuppressive effector pathways that have been extensively and elegantly characterized as crucial in models of MSC immunosuppression *in vitro* (75, 125, 126, 128, 132) have not been demonstrated to have a reproducible role in predicting MSC therapeutic activity *in vivo*. The impossibility to exploit these molecules as biomarkers should also be taken into account. Although it is relatively easy to determine and monitor these soluble molecules *in vitro*, our ability to measure them *in vivo* might be jeopardized by their restricted range of action and limited bioavailability over time. The correct timing to assess these effectors may also differ significantly based on the nature of studies (*in vitro* vs. *in vivo*), with a further layer of complexity associated with difference in metabolism secondary to patient age, disease severity, co-morbidities, or use of concomitant treatments.

At the state of the art, our well-established *in vitro* models of MSC immunosuppression are not fully elucidated for the development of robust biomarkers as predictors of clinical response in GvHD patients treated with MSCs. It appears that a better understanding of the mechanisms underlying MSC-mediated immunosuppression *in vivo* is therefore fundamental to provide novel perspectives and mechanistical platforms as starting points for the development of reliable biomarkers.

## Role of MSCs Undergoing Apoptosis to Deliver Immunosuppression *In vivo*

### MSCs Undergoing Apoptosis *in vivo* to Induce Immunosuppression

One major unresolved challenge which undermines the progress in our understanding of MSC immunosuppression *in vivo* is that the vast majority of infused MSCs become undetectable a few hours after transiently residing in the lungs (133, 134). Nevertheless, MSCs appear to maintain their ability to deliver therapeutic activities and engage with other regulatory cells like T-reg and macrophages. It is clear that our current *in vitro* models of MSC immunosuppression are still lacking some important aspects and cannot reconcile the paradox of the absence of engraftment and immunosuppressive functions (44, 135–137).

Starting from these observations, we tested the hypothesis that the lack of MSC engraftment might be due to cell death after infusion. In our experimental model of aGvHD, we have demonstrated that MSCs undergo extensive caspase activation and apoptosis after infusion in the presence of cytotoxic cells, and that this is a requirement for their immunosuppressive function (138). This apoptosis is mediated by both CD8 and NK cells and is not MHC-restricted. After MSC apoptosis, phagocytic cells are also required to engulf apoptotic MSCs and produce IDO which in turn triggers immunosuppression (138). These findings are in line with previous studies, whereby activated but not resting NK cells were able to lyse MSCs *in vitro* (46), or MSCs were cleared *in vivo* by deployment of different recipient-dependent reactions (139–143). Notably, these data provide a completely novel perspective which undermines the so-called “immune-privileged” status of MSCs. Conversely, by demonstrating the instrumental role of *in vivo* MSC apoptosis in delivering immunosuppression after infusion, they reconcile the role of the observed MSC rejection *in vivo* (144) in the context of MSC immunosuppressive functions across MHC barrier (72, 145) and highlight the capacity of apoptotic MSCs to modulate immune responses (146–149).

### MSC Apoptosis Provides a Predictive Biomarker Selecting MSC Responders in GvHD

The observation that MSC apoptosis requires and is induced by cytotoxic granules in a mouse model of aGvHD led us to investigate the role of cytotoxic immune cells against MSCs also in human patients. We found that the cytotoxic activity against MSCs can also be detected in the PBMCs of GvHD patients. More importantly, our data show that patients displaying high cytotoxicity respond to MSC therapy, whilst those with low or absent cytotoxic activity do not improve following MSC infusion (138). These data have now been confirmed in an extended cohort of patients and the cytotoxic assay has been found to predict clinical responses with high sensitivity and specificity (*Galleu A et al. Oral presentation, Abstract S252, EHA 2020*). It is important to point out that the limited number of patients analyzed warrants further validation in a prospective clinical study.

Currently, we do not know whether the cells mediating this cytotoxicity are derived from the donor of HSCT or from the recipient. Furthermore, it cannot be excluded that patients who have very poor reconstitution after HSCT with both CD8 and NK cells may have a hampered capacity to kill MSCs and then reduced likelihood to respond to MSC treatment. However, neither the absolute numbers nor the frequencies of CD8 and NK cells seem to have a role in predicting the response to MSCs (75), as supported by our observation that there is no difference between CD8 or NK cell percentages between responders and non-responders (138). It is likely that only a better characterization of the phenotype of the cytotoxic cells mediating MSC apoptosis will enable us to identify the actual subpopulation of cells eventually responsible of this apoptosis and to develop a quantitative and more approachable assay for use in a routine pathology laboratory.

Despite these limitations, this predictive biomarker represents a paradigm shift in MSC therapeutics. Its strength relies on the fact that the assay is supported by mechanistic insights. Furthermore, an important consequence of these observations is that, although MSCs remain the necessary starting point for therapeutic immunosuppression, patient-derived cells play a crucial role in delivering such an immunosuppression. This new perspective, in line with clinical data whereby MSCs from the same donor can give different responses in different patients (72–74, 76, 78, 79, 150), may significantly affect the Research and Development sector of MSC manufacturing. In the last decades, much efforts have been spent on the identification of the most clinically effective MSC preparations. Several strategies have been proposed, including the selection of MSCs based on biological parameters such as the magnitude of IDO synthesis (151) or the intracellular levels of the transcription factor TWIST1 (152). Conversely, other groups suggested to overcome the intrinsic variability among MSC batches by generating MSCs from pooled BM-MNCs of multiple third-party donors (153). It is not clear whether different MSCs exhibit similar or different capacity to undergo apoptosis. Further studies are needed to verify whether MSCs from different sources, administered after thawing or from fresh cultures, expanded in selected conditions, or differentially sorted based on specific features, have different susceptibility to undergo apoptosis. In this perspective, the cytotoxic assay may be devised as a tool for standardization of MSC manufacturing by select specific thresholds of killing used as product specification. Such an assay would also address the unmet need for a potency assay as a guideline for Regulatory Authority requirements (154) to implement quality control of manufactured MSCs. Thus far, most potency assays are designed with the aim to identify or select the “most immunosuppressive” MSC batches (155, 156), but they are exclusively based on MSC *in vitro* properties. By measuring their susceptibility to undergo apoptosis when exposed to cytotoxic cells, the cytotoxic assay would possibly identify “the most fit MSCs” which will deliver their therapeutic activity once administered to patients able to induce their apoptosis.

### MSC Apoptosis Provides a Monitoring Biomarker Evaluating MSC Immunological Effects in GvHD

The role of MSC apoptosis *in vivo* not only provides clinicians a powerful prognostic tool to predict patient responses to MSC treatment (138), it also paves the way for the development of potential tool to monitor the immunological effects after MSC infusion. The ground for this approach will be centered around the concept of the reprogramming of myeloid cells in the hosts following MSC apoptosis and efferocytosis. It has been well-documented that robust immune suppression and tolerance is mediated by myeloid cells (monocytes and dendritic cells in particular) following efferocytosis of apoptotic cells. These effects can be mediated by TGF-β (157, 158), IDO (147), IL-10 (159), or COX2/PGE2 (160). The field of dying MSCs has only begun to unveil their immunomodulation in certain models (161) and remains largely unexplored. However, it is conceivable that some of these factors might emerge as valuable biomarkers when further investigated.

In this perspective, the latest findings from our group seems to corroborate this idea. Indeed, we have demonstrated that efferocytosis of apoptotic MSCs endows monocytes with antiproliferative activity against T cells (162). These monocytes upregulated several immunosuppressive molecules, including metabolic enzymes IDO and COX2, immune checkpoint ligand PDL1 as well as soluble factors PGE2 (enzymatic product of COX2) and IL-10. Of note, the activity of COX2/PGE2 within the monocytes is in fact the key to determine the downstream expression of IDO, PDL1, and IL-10 as well as the monocyte inhibitory effects against T-cells. Most importantly, in a cohort of steroid-refractory aGvHD patients, the increase of serum PGE2 after MSC treatment is significantly higher in the responders compared to the non-responders. Hence, we suggest that the serum level of PGE2 can be evaluated as a biomarker for the monitoring of the immunological effects of MSCs in aGvHD patients receiving MSC treatment. PGE2 can be easily measured with the current biochemical methods such as ELISA, a rapid protocol with high sensitivity, specificity and reproducibility. With a reliable MSC monitoring biomarker, clinicians can be benefitted from an early predictor of treatment failure, thus promptly pursuing alternative treatments before the assessment of a response. Furthermore, this tool can be devised to optimize the MSC dosage or design a combinational regimen to improve the clinical efficacy of MSC therapy.

## Potential of Msc Extracellular Vesicles (EVs) as Biomarkers in GvHD

Besides the long-term notion about the importance of growth factors and cytokines as a part of the cell communication, the concept that cells also secrete large amounts of extracellular vesicles (EVs) as potential mediators is relatively new (163, 164). EVs are spherical structures limited by a lipid bilayer, which contains hydrophilic soluble components such as proteins, small and large RNA and DNA. There are different types of secreted EVs that have distinct structural and biochemical properties depending on their intracellular site of origin (165). Microvesicles and apoptotic bodies have been described as large EVs (>100 nm diameter) and can be formed at the plasma membrane by direct budding into the extracellular space. Smaller vesicles referred to as exosomes (around 100 nm diameter) are originated in multivesicular endosomes, subsequently secreted by fusion of these compartments with the plasma membrane (166).

The interest in EVs has progressively grown due to the discovery of their functional content, and the knowledge that the different EV subtypes contain molecules derived from different cellular compartments. Omics studies revealed that exosomes contain proteins originally located in the endosomes and microvesicles from cytosol and plasma membrane (167, 168). Apoptotic bodies, on the other hand, can contain molecules from the nucleus, endoplasmic reticulum or Golgi (169). Such a selectivity confirms that their cargos are not random, as might be in the case of cell debris. Instead, EVs contain a set of well-characterized and evolutionarily conserved proteins including the protein family of tetraspanins (CD63, CD81, and CD9) as well as Alix and TSG101, which have been used as EV markers. Also, they contain a set of molecules that varies according to different physiology, therefore understanding the EV cargo modifications, for instance during inflammation, may provide valuable insights into the prediction and/or monitoring of pathological processes (170).

EVs have been purified from many types of cell culture and are believed to be released from most, if not all, somatic cells, either constitutively or upon activation. Hence, they can be found in all different biological fluids such as plasma, serum, saliva and urine. Due to this specific content, EVs have been proposed as suitable biomarkers for various conditions (166). For instance, the use of EVs as a biomarker in allogenic transplantation context has been extensively investigated (171). In the study from Gunasekaran et al. (172) in lung transplantation, the detection of graft-derived exosomes preceded clinical diagnosis of graft rejection, suggesting that they could serve as a method to predict chronic rejection and adjust patient treatment accordingly. The predictive use of EVs as a biomarker was also suggested by Zhang et al. (173) In their study, they identified a panel of mRNAs (gp130, SH2D1B, TNFα, and CCL4) present in plasma EVs that could be used to predict on-going and/or imminent antibody-mediated rejection in kidney transplants. Other researchers found that they could correlate plasma and urinary EV content with graft rejection and its severity in renal (174–176) and lung transplanted patients (177). These results have shown that monitoring EVs and their cargos in patients might represent a promising non-invasive method to evaluate the status of allografts and the type and stage of rejection.

MSCs are well-characterized producers of a wide range of EVs with different cargos. The presence of selected miRNAs within MSC-derived EVs has been first described by Collino et al. (178). In their studies, they found that some of the miRNAs were present in both the EVs and the original cells. The similarities between cells and EVs were further confirmed by Kim et al. (179), when they conducted a study characterizing the protein content of BM-MSC-derived EVs and revealed their overlaps in surface markers, signaling molecules, cell adhesion molecules and additional MSC antigens. These data suggest MSC-derived EVs as potent mediators of intercellular communication locally and systemically.

EVs released from licensed MSCs have different composition and probably roles, when compared to those released by resting MSCs. Several studies have recently reported a significant variation on the EV content depending on the extracellular microenvironment priming the MSCs (180–183). Although the characterization of EVs released by the apoptotic MSCs is still under investigation, it is well-documented that apoptotic cells can produce a range of EVs and apoptotic bodies with different cargos that influences their microenvironment (169, 184, 185). In this regard, we can characterize the MSC-derived EVs to monitor their licensing process and/or the process of apoptosis. Furthermore, they might reveal the immunological effects of MSCs. Therefore, monitoring EVs isolated from the circulation of patients receiving MSCs holds a promising non-invasive method to evaluate the clinical efficacy of the treatment. Lastly, the analysis of EV content over time could also give hints of MSC kinetics, allowing to the adjustment of MSC administration accordingly.

## Conclusions

MSC immunobiology makes them ideal candidates for their use in cellular therapy in several immune mediated diseases, including GvHD. After thousands of infusions, the most convincing conclusion is that MSCs are well-tolerated and safe for patients. Major infectious events, secondary neoplasms, or malignancy relapse do not seem to increase after MSC therapy (33, 186). However, available data on MSC use in GvHD treatment represent the paradigm of the limitations of our current use of MSCs in most clinical applications. It is unquestionable that patients who responded to MSCs exhibit longer overall survivals than the non-responders (72–76, 78, 85). Importantly, this is a consistent finding across heterogenous cohorts of patients (Table 1). Nonetheless, there is not definitive and proved evidence of efficacy and responses are unpredictable.

The furious arguments ignited on the legitimacy of the use of MSCs in GvHD in the last few years highlights the unmet need to better understand how to improve the durability and the rates of responses to MSCs. We believe that only an in-depth understanding of the reasons behind clinical responses represents the necessary milestone for the design of the next generation of clinical trials in MSCs. Their success will undoubtedly route on our ability to identify the effective instruments (namely biomarkers and functional assays) that help us to predict clinical responses, guide us in selecting the best patient candidates, and ideally provide information as early predictors of treatment failure. Our ability to select only “fit patients” will be crucial in terms of sustainability of the costs of the MSC treatment and of a better management of limited resources, especially in the case of universal health care systems. The identification of such biomarkers will also harmonize the broad heterogeneity among MSC manufacturing processes across different centers (187). This will be a crucial pre-requisition for rigorous and scientific reproducibility across studies by which assess the difference between MSC preparations, MSC sources, administration regimens and doses.

**Figure 2:**
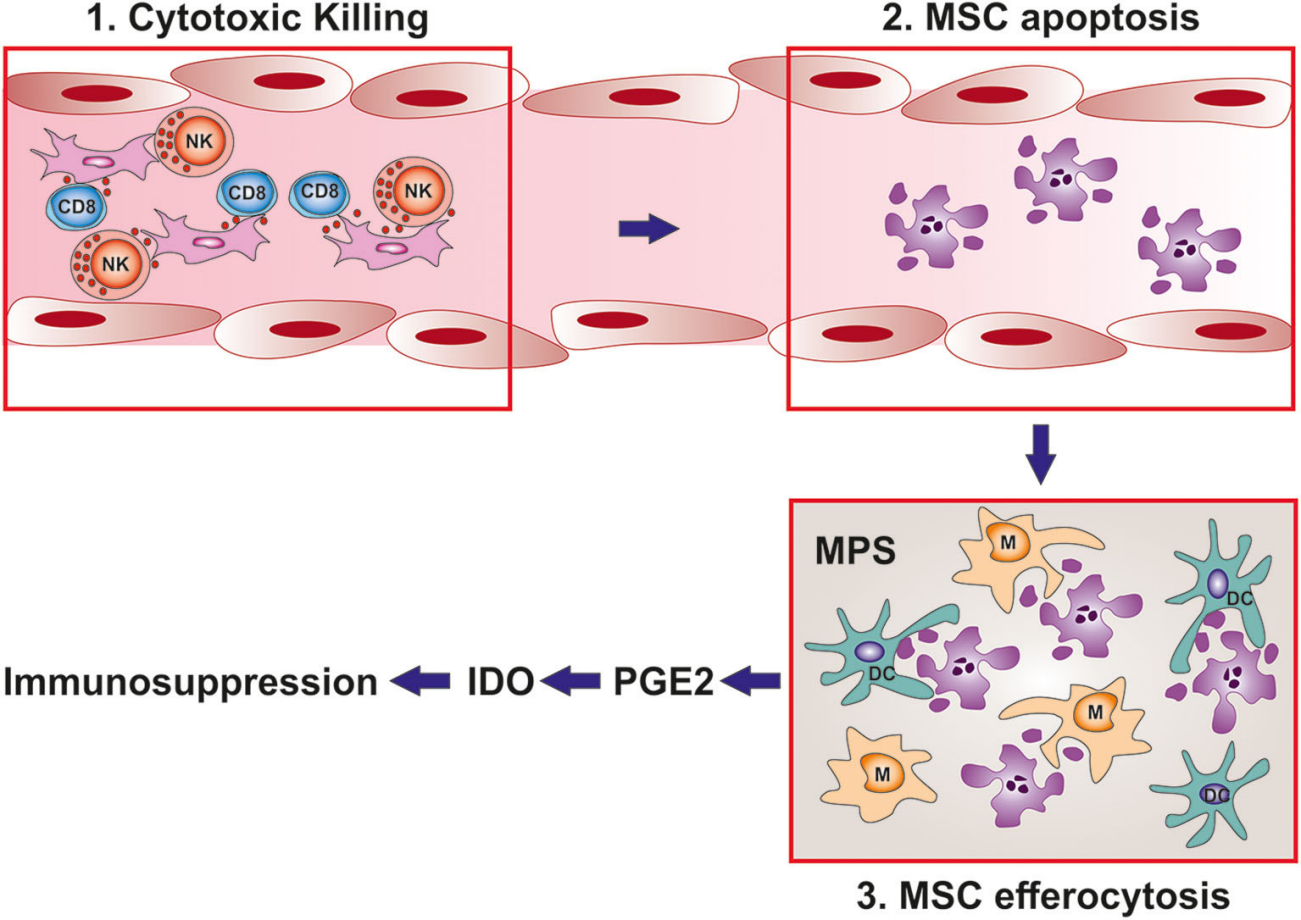
MSC immunomodulation depends on the interaction with the host. Schematic representation of MSC mediated immunosuppression after infusion. 1: After infusion, MSCs interact with the cytotoxic granules produced by CD8 T cells and NK cells of MSC recipient. 2: MSCs are induced to undergo apoptosis. 3: apoptotic MSCs are cleared from the circulation by the mononuclear phagocyte system. After efferocytosis, phagocytic cells of MSC recipient are induced to produce PGE2 and IDO which are the final mediator of MSC immunosuppression. Importantly, while the cytotoxic activity against MSC can be used as a biomarker to predict the response before MSC infusion, PGE2 levels in patient serum could be devised to monitor response after treatment.

So far, the translation of the *in vitro* models of MSC immunosuppression has failed to provide assays able to guide patient stratification. The discovery that MSC apoptosis is essential for MSC therapeutic efficacy *in vivo* represents a paradigm shift in the MSC field. This does not necessarily imply that it is the only possible mechanism and we cannot exclude the co-existence with other soluble-mediated mechanisms. However, this provides a reconciliation of the paradox between absence of engraftment and activity thus giving a strong mechanistic base of apparently contradictory experimental observations. Furthermore, in agreement with clinical data, it strengthens the notion that it is the patient with his/her inflammatory environment who plays a crucial role in the final response. Most importantly, this novel mechanism can be easily translated into reliable biomarkers. While the ability of the recipient to generate apoptotic MSCs appears to be a requirement for the therapeutic efficacy and could be used to stratify patients for MSC infusions before the treatment, the PGE2 levels in patient after MSC infusion could be exploited to monitor the response and provide a tool for detecting early treatment failures (Figure 2).

These new biomarkers may represent the dawn of a new era of MSC use in GvHD. However, we are only scratching the surface of the challenge in our attempt to improve the use of MSCs in GvHD and other inflammatory diseases. New questions need to be addressed and new paths identified to pave the way. Gaps are also yet to be filled regarding the relationship between MSC apoptosis and the classical “cytokine licensing.” A follow-on question regards the extent and the durability of the tolerogenic environment created by apoptotic MSCs. The restricted location of MSC apoptosis does not seem to reconcile with the systemic effects on inflammation. Answers to these questions will certainly provide novel insights and will lead us to the improvement of the available biomarkers or the discovery of new and more precise assays.

## Author Contributions

TC, GB, CG, FD, and AG designed the scope and context of this review. TC, GB, CG, and AG wrote the manuscript. TC, FD, and AG participated in manuscript revision and editing. MB, FD, and AG supervised the process. All authors contributed to the article and approved the submitted version.

## Conflict of Interest

The authors declare that the research was conducted in the absence of any commercial or financial relationships that could be construed as a potential conflict of interest.

## References

[1] Marigo I Dazzi F. The immunomodulatory properties of mesenchymal stem cells. Semin Immunopathol. (2011) 33:593–602. 10.1007/s00281-011-0267-7 21499984

[2] Friedenstein AJ Chailakhjan RK Lalykina KS. The development of fibroblast colonies in monolayer cultures of guinea-pig bone marrow and spleen cells. Cell Tissue Kinet. (1970) 3:393–403. 10.1111/j.1365-2184.1970.tb00347.x 5523063

[3] Friedenstein AJ Gorskaja JF Kulagina NN. Fibroblast precursors in normal and irradiated mouse hematopoietic organs. Exp Hematol. (1976) 4:267–274. 976387

[4] Caplan AI Mesenchymal stem cells. J Orthop Res. (1991) 9:641–50. 10.1002/jor.1100090504 1870029

[5] Kopen GC Prockop DJ Phinney DG. Marrow stromal cells migrate throughout forebrain and cerebellum, and they differentiate into astrocytes after. Proc Natl Acad Sci USA. (1999) 96:10711–6. 10.1073/pnas.96.19.10711 10485891 PMC17948

[6] da Silva Meirelles L Chagastelles PC Nardi NB. Mesenchymal stem cells reside in virtually all post-natal organs and tissues. J Cell Sci. (2006) 119:2204–13. 10.1242/jcs.02932 16684817

[7] Igura K Zhang X Takahashi K Mitsuru A Yamaguchi S Takahashi TA. Isolation and characterization of mesenchymal progenitor cells from chorionic villi of human placenta. Cytotherapy. (2004) 6:543–53. 10.1080/14653240410005366-1 15770794

[8] Erices A Conget P Minguell JJ. Mesenchymal progenitor cells in human umbilical cord blood. Br J Haematol. (2000) 109:235–42. 10.1046/j.1365-2141.2000.01986.x 10848804

[9] Zannettino ACW Paton S Arthur A Khor F Itescu S Gimble JM Gronthos S. Multipotential human adipose-derived stromal stem cells exhibit a perivascular phenotype *in vitro* and *in vivo*. J Cell Physiol. (2008) 214:413–21. 10.1002/jcp.21210 17654479

[10] Gronthos S Mankani M Brahim J Robey PG Shi S. Postnatal human dental pulp stem cells (DPSCs) *in vitro* and *invivo*. Proc Natl Acad Sci USA. (2000) 97:13625–30. 10.1073/pnas.240309797 11087820 PMC17626

[11] Pittenger MF Mackay AM Beck SC Jaiswal RK Douglas R Mosca JD. Multilineage potential of adult human mesenchymal stem cells. Science. (1999) 284:143–7. 10.1126/science.284.5411.143 10102814

[12] Haniffa MA Collin MP Buckley CD Dazzi F. Mesenchymal stem cells: The fibroblasts' new clothes? Haematologica. (2009) 94:258–63. 10.3324/haematol.13699 19109217 PMC2635401

[13] Sudo K Kanno M Miharada K Ogawa S Hiroyama T Saijo K Mesenchymal progenitors able to differentiate into osteogenic, chondrogenic, and/or adipogenic cells *in vitro* are present in most primary fibroblast-like cell populations. Stem Cells. (2007) 25:1610–7. 10.1634/stemcells.2006-0504 17395773

[14] Cheung TS Dazzi F. Mesenchymal-myeloid interaction in the regulation of immunity. Semin Immunol. (2018) 35:59–68. 10.1016/j.smim.2018.01.002 29395680

[15] Hass R Kasper C Böhm S Jacobs R. Different populations and sources of human mesenchymal stem cells (MSC): a comparison of adult and neonatal tissue-derived MSC. Cell Commun Signal. (2011) 9:12. 10.1186/1478-811X-9-12 21569606 PMC3117820

[16] Noort W Scherjon S Kleijburg-van der Keur C Kruisselbrink A van Bezooijen R Beekhuizen W. Mesenchymal stem cells in human second-trimester bone marrow, liver, lung, and spleen exhibit a similar immunophenotype but a heterogeneous multilineage differentiation potential. Haematologica. (2003) 88:845–52. 12935972

[17] Bieback K Kern S Kocaömer A Ferlik K Bugert P. Comparing mesenchymal stromal cells from different human tissues: bone marrow, adipose tissue and umbilical cord blood. Biomed Mater Eng. (2008) 18:S71–6. 18334717

[18] Mets T Verdonk G. *in vitro* aging of human bone marrow derived stromal cells. Mech Ageing Dev. (1981) 16:81–9. 10.1016/0047-6374(81)90035-X 7253722

[19] Yang YHK Ogando CR Wang See C Chang TY Barabino GA. Changes in phenotype and differentiation potential of human mesenchymal stem cells aging *in vitro*. Stem Cell Res Ther. (2018) 9:1–14. 10.1186/s13287-018-0876-3 29751774 PMC5948736

[20] Tse WT Pendleton JD Beyer WM Egalka MC Guinan EC. Suppression of allogeneic T-cell proliferation by human marrow stromal cells: implications in transplantation. Transplantation. (2003) 75:389–97. 10.1097/01.TP.0000045055.63901.A9 12589164

[21] Rasmusson I Ringdén O Sundberg B Le Blanc K Mesenchymal stem cells inhibit the formation of cytotoxic T lymphocytes, but not activated cytotoxic T lymphocytes or natural killer cells. Transplantation. (2003) 76:1208–13. 10.1097/01.TP.0000082540.43730.80 14578755

[22] Bartholomew A Sturgeon C Siatskas M Ferrer K McIntosh K Patil S. Mesenchymal stem cells suppress lymphocyte proliferation *in vitro* and prolong skin graft survival *in vivo*. Exp Hematol. (2002) 30:42–8. 10.1016/S0301-472X(01)00769-X 11823036

[23] Horwitz EM Gordon PL Koo WKK Marx JC Neel MD McNall RY. Isolated allogeneic bone marrow-derived mesenchymal cells engraft and stimulate growth in children with osteogenesis imperfecta: Implications for cell therapy of bone. Proc Natl Acad Sci USA. (2002) 99:8932–7. 10.1073/pnas.132252399 12084934 PMC124401

[24] Al-Khaldi A Al-Sabti H Galipeau J Lachapelle K. Therapeutic angiogenesis using autologous bone marrow stromal cells: Improved blood flow in a chronic limb ischemia model. Ann Thorac Surg. (2003) 75:204–9. 10.1016/S0003-4975(02)04291-1 12537217

[25] Burr SP Dazzi F Garden OA. Mesenchymal stromal cells and regulatory T cells: the Yin and Yang of peripheral tolerance? Immunol Cell Biol. (2013) 91:12–8. 10.1038/icb.2012.60 23146942

[26] Bernardo ME Fibbe WE. Safety and efficacy of mesenchymal stromal cell therapy in autoimmune disorders. Ann N Y Acad Sci. (2012) 1266:107–17. 10.1111/j.1749-6632.2012.06667.x 22901262

[27] Duffy MM Ritter T Ceredig R Griffin MD. Mesenchymal stem cell effects on T-cell effector pathways. Stem Cell Res Ther. (2011) 2:34. 10.1186/scrt75 21861858 PMC3219065

[28] Aggarwal S Pittenger MF. Human mesenchymal stem cells modulate allogeneic immune cell responses. Blood. (2005) 105:1815–22. 10.1182/blood-2004-04-1559 15494428

[29] Bianco P Cao X Frenette PS Mao JJ Robey PG Simmons PJ. The meaning, the sense and the significance: translating the science of mesenchymal stem cells into medicine. Nat Med. (2013) 19:35–42. 10.1038/nm.3028 23296015 PMC3998103

[30] Caplan AI. Mesenchymal stem cells: time to change the name! Stem Cells Transl Med. (2017) 6:1445–51. 10.1002/sctm.17-0051 28452204 PMC5689741

[31] Polchert D Sobinsky J Douglas G Kidd M Moadsiri A Reina E. IFN-gamma activation of mesenchymal stem cells for treatment and prevention of graft versus host disease. Eur J Immunol. (2008) 38:1745–55. 10.1002/eji.200738129 18493986 PMC3021120

[32] Tisato V Naresh K Girdlestone J Navarrete C Dazzi F. Mesenchymal stem cells of cord blood origin are effective at preventing but not treating graft-versus-host disease. Leukemia. (2007) 21:1992–9. 10.1038/sj.leu.2404847 17625609

[33] Wang L Zhu C Ma D Gu Z Xu C Wang F. Efficacy and safety of mesenchymal stromal cells for the prophylaxis of chronic graft-versus-host disease after allogeneic hematopoietic stem cell transplantation: a meta-analysis of randomized controlled trials. Ann Hematol. (2018) 97:1941–50. 10.1007/s00277-018-3384-8 29947972

[34] Spaggiari GM Capobianco A Abdelrazik H Becchetti F Mingari MC Moretta L. Mesenchymal stem cells inhibit natural killer-cell proliferation, cytotoxicity, and cytokine production: Role of indoleamine 2,3-dioxygenase and prostaglandin E2. Blood. (2008) 111:1327–33. 10.1182/blood-2007-02-074997 17951526

[35] Ryan JM Barry F Murphy JM Mahon BP. Interferon-γ does not break, but promotes the immunosuppressive capacity of adult human mesenchymal stem cells. Clin Exp Immunol. (2007) 149:353–63. 10.1111/j.1365-2249.2007.03422.x 17521318 PMC1941956

[36] Krampera M Cosmi L Angeli R Pasini A Liotta F Andreini A. Role for interferon-gamma in the immunomodulatory activity of human bone marrow mesenchymal stem cells. Stem Cells. (2006) 24:386–98. 10.1634/stemcells.2005-0008 16123384

[37] Chabannes D Hill M Merieau E Rossignol J Brion R Soulillou JP. A role for heme oxygenase-1 in the immunosuppressive effect of adult rat and human mesenchymal stem cells. Blood. (2007) 110:3691–4. 10.1182/blood-2007-02-075481 17684157

[38] Nasef A Chapel A Mazurier C Bouchet S Lopez M Mathieu N. Identification of IL-10 and TGF-β transcripts Involved in the Inhibition of T-Lymphocyte proliferation during cell contact with human mesenchymal stem cells. Gene Expr. (2018) 13:217–26. 10.3727/000000006780666957 17605296 PMC6032462

[39] Selmani Z Naji A Zidi I Favier B Gaiffe E Obert L. Human leukocyte antigen-G5 secretion by human mesenchymal stem cells is required to suppress T lymphocyte and natural killer function and to induce CD4+CD25highFOXP3+ regulatory T cells. Stem Cells. (2008) 26:212–22. 10.1634/stemcells.2007-0554 17932417

[40] Nasef A Mazurier C Bouchet S François S Chapel A Thierry D. Leukemia inhibitory factor: role in human mesenchymal stem cells mediated immunosuppression. Cell Immunol. (2008) 253:16–22. 10.1016/j.cellimm.2008.06.002 18639869

[41] Gieseke F Böhringer J Bussolari R Dominici M Handgretinger R Dc W. Immune effector cells human multipotent mesenchymal stromal cells use galectin-1 to inhibit immune effector cells. Blood. (2012) 116:3770–9. 10.1182/blood-2010-02-270777 20644118

[42] Sioud M Mobergslien A Boudabous A Fløisand Y. Evidence for the involvement of galectin-3 in mesenchymal stem cell suppression of allogeneic T-cell proliferation. Scand J Immunol. (2010) 71:267–74. 10.1111/j.1365-3083.2010.02378.x 20384870

[43] Ungerer C Quade-Lyssy P Radeke HH Henschler R Königs C Köhl U. Galectin-9 is a suppressor of T and B cells and predicts the immune modulatory potential of mesenchymal stromal cell preparations. Stem Cells Dev. (2014) 23:755–66. 10.1089/scd.2013.0335 24083426 PMC3967371

[44] Glennie S Soeiro I Dyson PJ Lam EW Dazzi F. Bone marrow mesenchymal stem cells induce division arrest anergy of activated T cells. Blood. (2005) 105:2821–7. 10.1182/blood-2004-09-3696 15591115

[45] Sotiropoulou PA Perez SA Gritzapis AD Baxevanis CN Papamichail M. Interactions between human mesenchymal stem cells and natural killer cells. Stem Cells. (2006) 24:74–85. 10.1634/stemcells.2004-0359 16099998

[46] Spaggiari GM Capobianco A Becchetti S Mingari MC Moretta L. Mesenchymal stem cell-natural killer cell interactions: evidence that activated NK cells are capable of killing MSCs, whereas MSCs can inhibit IL-2-induced NK-cell proliferation. Blood. (2006) 107:1484–90. 10.1182/blood-2005-07-2775 16239427

[47] Augello A Tasso R Negrini S Amateis A Indiveri F Cancedda R. Bone marrow mesenchymal progenitor cells inhibit lymphocyte proliferation by activation of the programmed death 1 pathway. Eur J Immunol. (2005) 35:1482–90. 10.1002/eji.200425405 15827960

[48] Corcione A Benvenuto F Ferretti E Giunti D Cappiello V Cazzanti F. Human mesenchymal stem cells modulate B-cell functions. Blood. (2006) 107:367–72. 10.1182/blood-2005-07-2657 16141348

[49] Sheng H Wang Y Jin Y Zhang Q Zhang Y Wang L. A critical role of IFNγ in priming MSC-mediated suppression of T cell proliferation through up-regulation of B7-H1. Cell Res. (2008) 18:846–57. 10.1038/cr.2008.80 18607390

[50] Tipnis S Viswanathan C Majumdar AS. Immunosuppressive properties of human umbilical cord-derived mesenchymal stem cells: role of B7-H1 and IDO. Immunol Cell Biol. (2010) 88:795–806. 10.1038/icb.2010.47 20386557

[51] Giuliani M Fleury M Vernochet A Ketroussi F Clay D Azzarone B. Long-lasting inhibitory effects of fetal liver mesenchymal stem cells on T-lymphocyte proliferation. PLoS ONE. (2011) 6:e19988. 10.1371/journal.pone.0019988 21625521 PMC3098287

[52] Shi C Jia T Mendez-Ferrer S Hohl TM Serbina NV Lipuma L. Bone marrow mesenchymal stem and progenitor cells induce monocyte emigration in response to circulating toll-like receptor ligands. Immunity. (2011) 34:590–601. 10.1016/j.immuni.2011.02.016 21458307 PMC3081416

[53] Miteva K Pappritz K El-Shafeey M Dong F Ringe J Tschöpe C. Mesenchymal Stromal cells modulate monocytes trafficking in coxsackievirus B3-induced myocarditis. Stem Cells Transl Med. (2017) 6:1249–61. 10.1002/sctm.16-0353 28186704 PMC5442851

[54] Kim J Hematti P. Mesenchymal stem cell-educated macrophages: a novel type of alternatively activated macrophages. Exp Hematol. (2009) 37:1445–53. 10.1016/j.exphem.2009.09.004 19772890 PMC2783735

[55] Trento C Marigo I Pievani A Galleu A Dolcetti L Wang CY. Bone marrow mesenchymal stromal cells induce nitric oxide synthase-dependent differentiation of CD11b+ cells that expedite hematopoietic recovery. Haematologica. (2017) 102:818–25. 10.3324/haematol.2016.155390 28183849 PMC5477600

[56] Jiang XX Zhang Y Liu B Zhang SX Wu Y Yu XD. Human mesenchymal stem cells inhibit differentiation and function of monocyte-derived dendritic cells. Blood. (2005) 105:4120–6. 10.1182/blood-2004-02-0586 15692068

[57] Ramasamy R Fazekasova H Lam EWF Soeiro I Lombardi G Dazzi F. Mesenchymal stem cells inhibit dendritic cell differentiation and function by preventing entry into the cell cycle. Transplantation. (2007) 83:71–6. 10.1097/01.tp.0000244572.24780.54 17220794

[58] Zhang B Liu R Shi D Liu X Chen Y Dou X. Mesenchymal stem cells induce mature dendritic cells into a novel Jagged-2-dependent regulatory dendritic cell population. Blood. (2009) 113:46–57. 10.1182/blood-2008-04-154138 18832657

[59] Cahill EF Tobin LM Carty F Mahon BP English K. Jagged-1 is required for the expansion of CD4+ CD25+ FoxP3+ regulatory T cells and tolerogenic dendritic cells by murine mesenchymal stromal cells. Stem Cell Res Ther. (2015) 6:19. 10.1186/s13287-015-0021-5 25890330 PMC4414370

[60] Maccario R Podestà M Moretta A Cometa A Comoli P Montagna D. Interaction of human mesenchymal stem cells with cells involved in alloantigen-specific immune response favors the differentiation of CD4+ T-cell subsets expressing a regulatory/suppressive phenotype. Haematologica. (2005) 90:516–25. 15820948

[61] Kavanagh H Mahon BP. Allogeneic mesenchymal stem cells prevent allergic airway inflammation by inducing murine regulatory T cells. Allergy. (2011) 66:523–31. 10.1111/j.1398-9995.2010.02509.x 21091718

[62] Luz-Crawford P Kurte M Bravo-Alegría J Contreras R Nova-Lamperti E Tejedor G. Mesenchymal stem cells generate a CD4+CD25+Foxp3+ regulatory T cell population during the differentiation process of Th1 and Th17 cells. Stem Cell Res Ther. (2013) 4:65. 10.1186/scrt216 23734780 PMC3706898

[63] Ge W Jiang J Arp J Liu W Garcia B Wang H. Regulatory T-cell generation and kidney allograft tolerance induced by mesenchymal stem cells associated with indoleamine 2,3-dioxygenase expression. Transplantation. (2010) 90:1312–20. 10.1097/TP.0b013e3181fed001 21042238

[64] Melief SM Schrama E Brugman MH Tiemessen MM Hoogduijn MJ Fibbe WE. Multipotent stromal cells induce human regulatory T cells through a novel pathway involving skewing of monocytes toward anti-inflammatory macrophages. Stem Cells. (2013) 31:1980–91. 10.1002/stem.1432 23712682

[65] Haniffa MA Wang X Holtick U Rae M Isaacs JD Dickinson AM. Adult human fibroblasts are potent immunoregulatory cells and functionally equivalent to mesenchymal stem cells. J Immunol. (2007) 179:1595–604. 10.4049/jimmunol.179.3.1595 17641026

[66] Ren G Zhang L Zhao X Xu G Zhang Y Roberts AI. Mesenchymal stem cell-mediated immunosuppression occurs via concerted action of chemokines and nitric oxide. Cell Stem Cell. (2008) 2:141–50. 10.1016/j.stem.2007.11.014 18371435

[67] Ferrara JL Levine JE Reddy P Holler E. Graft-versus-host disease. Lancet. (2009) 373:1550–61. 10.1016/S0140-6736(09)60237-3 19282026 PMC2735047

[68] Welniak LA Blazar BR Murphy WJ. Immunobiology of allogeneic hematopoietic stem cell transplantation. Annu Rev Immunol. (2007) 25:139–70. 10.1146/annurev.immunol.25.022106.141606 17129175

[69] Filipovich AH Weisdorf D Pavletic S Socie G Wingard JR Lee SJ National institutes of health consensus development project on criteria for clinical trials in chronic graft-versus-host disease: I. Diagnosis and staging working group report. Biol Blood Marrow Transplant. (2005) 11:945–56. 10.1016/j.bbmt.2005.09.004 16338616

[70] Jagasia MH Greinix HT Arora M Williams KM Wolff D Cowen EW National institutes of health consensus development project on criteria for clinical trials in chronic graft-versus-host disease: I. The 2014 diagnosis and staging working group report. Biol Blood Marrow Transplant. (2015) 21:389–401.e1. 10.1016/j.bbmt.2014.12.001 25529383 PMC4329079

[71] Jamani K Russell JA Daly A Stewart D Savoie L Duggan P. Prognosis of grade 3-4 acute GVHD continues to be dismal. Bone Marrow Transplant. (2013) 48:1359–61. 10.1038/bmt.2013.59 23604011

[72] Le Blanc K Frassoni F Ball L Locatelli F Roelofs H Lewis I. Mesenchymal stem cells for treatment of steroid-resistant, severe, acute graft-versus-host disease: a phase II study. Lancet. (2008) 371:1579–86. 10.1016/S0140-6736(08)60690-X 18468541

[73] Ball LM Bernardo ME Roelofs H van Tol MJ Contoli B Zwaginga JJ. Multiple infusions of mesenchymal stromal cells induce sustained remission in children with steroid-refractory, grade III-IV acute graft-versus-host disease. Br J Haematol. (2013) 163:501–9. 10.1111/bjh.12545 23992039

[74] Kurtzberg J Prockop S Teira P Bittencourt H Lewis V Chan KW. Allogeneic human mesenchymal stem cell therapy (remestemcel-L, Prochymal) as a rescue agent for severe refractory acute graft-versus-host disease in pediatric patients. Biol Blood Marrow Transpl. (2014) 20:229–35. 10.1016/j.bbmt.2013.11.001 24216185

[75] Te Boome LCJ Mansilla C Van Der Wagen LE Lindemans CA Petersen EJ Spierings E. Biomarker profiling of steroid-resistant acute GVHD in patients after infusion of mesenchymal stromal cells. Leukemia. (2015) 29:1839–46. 10.1038/leu.2015.89 25836589

[76] Kebriaei P Isola L Bahceci E Holland K Rowley S McGuirk J. Adult human mesenchymal stem cells added to corticosteroid therapy for the treatment of acute graft-versus-host disease. Biol Blood Marrow Transpl. (2009) 15:804–11. 10.1016/j.bbmt.2008.03.012 19539211

[77] Erbey F Atay D Akcay A Ovali E Ozturk G. Mesenchymal stem cell treatment for steroid refractory graft-versus-host disease in children: a pilot and first study from Turkey. Stem Cells Int. (2016) 2016:1–6. 10.1155/2016/1641402 26783400 PMC4691494

[78] Servais S Baron F Lechanteur C Seidel L Selleslag D Maertens J. Infusion of bone marrow derived multipotent mesenchymal stromal cells for the treatment of steroid-refractory acute graft-versus-host disease: a multicenter prospective study. Oncotarget. (2018) 9:20590–604. 10.18632/oncotarget.25020 29755674 PMC5945536

[79] von Dalowski F Kramer M Wermke M Wehner R Rollig C Alakel N. Mesenchymal stromal cells for treatment of acute steroid-refractory graft versus host disease: clinical responses and long-term outcome. Stem Cells. (2016) 34:357–66. 10.1002/stem.2224 26418955

[80] Dotoli GM De Santis GC Orellana MD de Lima Prata K Caruso SR Fernandes TR. Mesenchymal stromal cell infusion to treat steroid-refractory acute GvHD III/IV after hematopoietic stem cell transplantation. Bone Marrow Transplant. (2017) 52:859–62. 10.1038/bmt.2017.35 28287644

[81] Bader P Kuçi Z Bakhtiar S Basu O Bug G Dennis M. Effective treatment of steroid and therapy-refractory acute graft-versus-host disease with a novel mesenchymal stromal cell product (MSC-FFM). Bone Marrow Transplant. (2018) 53:852–62. 10.1038/s41409-018-0102-z 29379171 PMC6039391

[82] Introna M Lucchini G Dander E Galimberti S Rovelli A Balduzzi A. Treatment of graft versus host disease with mesenchymal stromal cells: a phase I study on 40 adult and pediatric patients. Biol Blood Marrow Transplant. (2014) 20:375–81. 10.1016/j.bbmt.2013.11.033 24321746

[83] Fernández-Maqueda C Gonzalo-Daganzo R Regidor C Martín-Donaire T Sánchez R Bueno JL. Mesenchymal stromal cells for steroid-refractory acute GvHD. Bone Marrow Transplant. (2017) 52:1577–9. 10.1038/bmt.2017.177 28783146

[84] Resnick IB Barkats C Shapira MY Stepensky P Bloom AI Shimoni A. Treatment of severe steroid resistant acute GVHD with mesenchymal stromal cells (MSC). Am J Blood Res. (2013) 3:225–38. 23997985 PMC3755522

[85] Galleu A Milojkovic D Deplano S Szydlo R Loaiza S Wynn R. Mesenchymal stromal cells for acute graft-versus-host disease: response at 1 week predicts probability of survival. Br J Haematol. (2019) 185:89–92. 10.1111/bjh.15749 30637732 PMC6916615

[86] Wu KH Chan CK Tsai C Chang YH Sieber M Chiu TH. Effective treatment of severe steroid-resistant acute graft-versus-host disease with umbilical cord-derived mesenchymal stem cells. Transplantation. (2011) 91:1412–6. 10.1097/TP.0b013e31821aba18 21494176

[87] Chen GH Yang T Tian H Qiao M Liu HW Fu CC. [Clinical study of umbilical cord-derived mesenchymal stem cells for treatment of nineteen patients with steroid-resistant severe acute graft-versus-host disease]. Zhonghua Xue Ye Xue Za Zhi. (2012) 33:303–6. 10.1016/j.jcyt.2014.01.114 22781723

[88] Fang B Song Y Lin Q Zhang Y Cao Y Zhao RC. Human adipose tissue-derived mesenchymal stromal cells as salvage therapy for treatment of severe refractory acute graft-vs.-host disease in two children. Pediatr Transpl. (2007) 11:814–7. 10.1111/j.1399-3046.2007.00780.x 17910665

[89] Fang B Song Y Liao L Zhang Y Zhao RC. Favorable response to human adipose tissue-derived mesenchymal stem cells in steroid-refractory acute graft-versus-host disease. Transpl Proc. (2007) 39:3358–62. 10.1016/j.transproceed.2007.08.103 18089385

[90] Ringden O Baygan A Remberger M Gustafsson B Winiarski J Khoein B. Placenta-derived decidua stromal cells for treatment of severe acute graft-versus-host disease. Stem Cells Transl Med. (2018) 7:325–31. 10.1002/sctm.17-0167 29533533 PMC5866941

[91] Yoo KH Jang IK Lee MW Kim HE Yang MS Eom Y. Comparison of immunomodulatory properties of mesenchymal stem cells derived from adult human tissues. Cell Immunol. (2009) 259:150–6. 10.1016/j.cellimm.2009.06.010 19608159

[92] Friedman R Betancur M Boissel L Tuncer H Cetrulo C Klingemann H. Umbilical cord mesenchymal stem cells: adjuvants for human cell transplantation. Biol Blood Marrow Transpl. (2007) 13:1477–86. 10.1016/j.bbmt.2007.08.048 18022578

[93] Roy S Arora S Kumari P Ta M. A simple and serum-free protocol for cryopreservation of human umbilical cord as source of Wharton's jelly mesenchymal stem cells. Cryobiology. (2014) 68:467–72. 10.1016/j.cryobiol.2014.03.010 24704519

[94] Kern S Eichler H Stoeve J Kluter H Bieback K Comparative analysis of mesenchymal stem cells from bone marrow, umbilical cord blood, or adipose tissue. Stem Cells. (2006) 24:1294–301. 10.1634/stemcells.2005-0342 16410387

[95] Rebelatto CK Aguiar AM Moretao MP Senegaglia AC Hansen P Barchiki F. Dissimilar differentiation of mesenchymal stem cells from bone marrow, umbilical cord blood, and adipose tissue. Exp Biol Med. (2008) 233:901–13. 10.3181/0712-RM-356 18445775

[96] Salmenniemi U Itälä-Remes M Nystedt J Putkonen M Niittyvuopio R Vettenranta K. Good responses but high TRM in adult patients after MSC therapy for GvHD. Bone Marrow Transplant. (2017) 52:606–8. 10.1038/bmt.2016.317 27941780

[97] Herrmann R Sturm M Shaw K Purtill D Cooney J Wright M. Mesenchymal stromal cell therapy for steroid-refractory acute and chronic graft versus host disease: a phase 1 study. Int J Hematol. (2012) 95:182–8. 10.1007/s12185-011-0989-2 22183779

[98] Perez-Simon JA Lopez-Villar O Andreu EJ Rifon J Muntion S Campelo MD. Mesenchymal stem cells expanded *in vitro* with human serum for the treatment of acute and chronic graft-versus-host disease: results of a phase I/II clinical trial. Haematologica. (2011) 96:1072–6. 10.3324/haematol.2010.038356 21393326 PMC3128230

[99] Lucchini G Introna M Dander E Rovelli A Balduzzi A Bonanomi S. Platelet-lysate-expanded mesenchymal stromal cells as a salvage therapy for severe resistant graft-versus-host disease in a pediatric population. Biol Blood Marrow Transplant. (2010) 16:1293–301. 10.1016/j.bbmt.2010.03.017 20350611

[100] Zhou H Guo M Bian C Sun Z Yang Z Zeng Y. Efficacy of bone marrow-derived mesenchymal stem cells in the treatment of sclerodermatous chronic graft-versus-host disease: clinical report. Biol Blood Marrow Transpl. (2010) 16:403–12. 10.1016/j.bbmt.2009.11.006 19925878

[101] Peng Y Chen X Liu Q Xu D Zheng H Liu L. Alteration of naïve and memory B-cell subset in chronic graft-versus-host disease patients after treatment with mesenchymal stromal cells. Stem Cells Transl Med. (2014) 3:1023–31. 10.5966/sctm.2014-0001 25015640 PMC4149298

[102] Weng JY Du X Geng SX Peng YW Wang Z Lu ZS. Mesenchymal stem cell as salvage treatment for refractory chronic GVHD. Bone Marrow Transpl. (2010) 45:1732–40. 10.1038/bmt.2010.195 20818445 PMC3035976

[103] Jurado M De La Mata C Ruiz-García A López-Fernández E Espinosa O Remigia MJ. Adipose tissue-derived mesenchymal stromal cells as part of therapy for chronic graft-versus-host disease: A phase I/II study. Cytotherapy. (2017) 19:927–36. 10.1016/j.jcyt.2017.05.002 28662983

[104] Koc ON Gerson SL Cooper BW Dyhouse SM Haynesworth SE Caplan AI. Rapid hematopoietic recovery after coinfusion of autologous-blood stem cells and culture-expanded marrow mesenchymal stem cells in advanced breast cancer patients receiving high-dose chemotherapy. J Clin Oncol. (2000) 18:307–16. 10.1200/JCO.2000.18.2.307 10637244

[105] Le Blanc K Samuelsson H Gustafsson B Remberger M Sundberg B Arvidson J. Transplantation of mesenchymal stem cells to enhance engraftment of hematopoietic stem cells. Leukemia. (2007) 21:1733–8. 10.1038/sj.leu.2404777 17541394

[106] Lazarus HM Koc ON Devine SM Curtin P Maziarz RT Holland HK. Cotransplantation of HLA-identical sibling culture-expanded mesenchymal stem cells and hematopoietic stem cells in hematologic malignancy patients. Biol Blood Marrow Transplant. (2005) 11:389–98. 10.1016/j.bbmt.2005.02.001 15846293

[107] Ball LM Bernardo ME Roelofs H Lankester A Cometa A Egeler RM. Cotransplantation of *ex vivo* expanded mesenchymal stem cells accelerates lymphocyte recovery and may reduce the risk of graft failure in haploidentical hematopoietic stem-cell transplantation. Blood. (2007) 110:2764–7. 10.1182/blood-2007-04-087056 17638847

[108] Li H Guo Z Jiang X Zhu H Li X Mao N. Mesenchymal stem cells alter migratory property of T and dendritic cells to delay the development of murine lethal acute graft-versus-host disease. Stem Cells. (2008) 26:2531–41. 10.1634/stemcells.2008-0146 18635870

[109] Li H Guo Z Zhu H Li XS Jiang X Yao H. Transplanted mesenchymal stem cells can inhibit the three developmental stages of murine acute graft-versus-host disease. In Vivo. (2010) 24:659–66. 20952730

[110] Macmillan ML Blazar BR DeFor TE Wagner JE. Transplantation of *ex-vivo* culture-expanded parental haploidentical mesenchymal stem cells to promote engraftment in pediatric recipients of unrelated donor umbilical cord blood: results of a phase I-II clinical trial. Bone Marrow Transpl. (2009) 43:447–54. 10.1038/bmt.2008.348 18955980

[111] Poloni A Leoni P Buscemi L Balducci F Pasquini R Masia MC. Engraftment capacity of mesenchymal cells following hematopoietic stem cell transplantation in patients receiving reduced-intensity conditioning regimen. Leukemia. (2006) 20:329–35. 10.1038/sj.leu.2404018 16341047

[112] Wu Y Wang Z Cao Y Xu L Li X Liu P. Cotransplantation of haploidentical hematopoietic and umbilical cord mesenchymal stem cells with a myeloablative regimen for refractory/relapsed hematologic malignancy. Ann Hematol. (2013) 92:1675–84. 10.1007/s00277-013-1831-0 23842707

[113] Wu Y Cao Y Li X Xu L Wang Z Liu P. Cotransplantation of haploidentical hematopoietic and umbilical cord mesenchymal stem cells for severe aplastic anemia: successful engraftment and mild GVHD. Stem Cell Res. (2014) 12:132–8. 10.1016/j.scr.2013.10.001 24185180

[114] Bernardo ME Ball LM Cometa AM Roelofs H Zecca M Avanzini MA Co-infusion of *ex vivo*-expanded, parental MSCs prevents life-threatening acute GVHD, but does not reduce the risk of graft failure in pediatric patients undergoing allogeneic umbilical cord blood transplantation. Bone Marrow Transpl. (2011) 46:200–7. 10.1038/bmt.2010.87 20400983

[115] Gonzalo-Daganzo R Regidor C Martin-Donaire T Rico MA Bautista G Krsnik I. Results of a pilot study on the use of third-party donor mesenchymal stromal cells in cord blood transplantation in adults. Cytotherapy. (2009) 11:278–88. 10.1080/14653240902807018 19308773

[116] Kallekleiv M Larun L Bruserud Ø Hatfield KJ. Co-transplantation of multipotent mesenchymal stromal cells in allogeneic hematopoietic stem cell transplantation: a systematic review and meta-analysis. Cytotherapy. (2016) 18:172–85. 10.1016/j.jcyt.2015.11.010 26794711

[117] Szabolcs P Visani G Locatelli F Kleiner G Talano J Nemecek E Treatment of steroid-refractory acute GVHD with mesenchymal stem cells improves outcomes in pediatric patients; results of the pediatric subset in a phase III randomized, placebo-controlled study. Biol Blood Marrow Transplant. (2010) 16:S298 10.1016/j.bbmt.2009.12.426

[118] Martin PJ Uberti JP Soiffer RJ Klingemann H Waller EK Daly AS Prochymal improves response rates in patients with steroid-refractory acute graft versus host disease (SR-GVHD) involving the liver and gut: results of a randomized, placebo-controlled, multicenter phase III trial in GVHD. Biol Blood Marrow Transplant. (2010) 16:S169–70. 10.1016/j.bbmt.2009.12.057

[119] Galipeau J The mesenchymal stromal cells dilemma–does a negative phase III trial of random donor mesenchymal stromal cells in steroid-resistant graft-versus-host disease represent a death knell or a bump in the road? Cytotherapy. (2013) 15:2–8. 10.1016/j.jcyt.2012.10.002 23260081

[120] NHS England Specialised Services Clinical Reference Group, Transplantation B and M Clinical commissioning policy: treatments for graft versus host disease (GvHD) following haematopoietic stem cell transplantation (2017) 16069/P. Available online at: https://www.england.nhs.uk/wp-content/uploads/2017/03/gvhd-heamatopoietic-stem-cell.pdf

[121] Biomarkers Definitions Working Group. Biomarkers and surrogate endpoints: Preferred definitions and conceptual framework. Clin Pharmacol Ther. (2001) 69:89–95. 10.1067/mcp.2001.113989 11240971

[122] Paczesny S Krijanovski OI Braun TM Choi SW Clouthier SG Kuick R. A biomarker panel for acute graft-versus-host disease. Blood. (2009) 113:273–8. 10.1182/blood-2008-07-167098 18832652 PMC2615645

[123] Ferrara JLM Harris AC Greenson JK Braun TM Holler E Teshima T. Regenerating islet-derived 3-alpha is a biomarker of gastrointestinal graft-versus-host disease. Blood. (2011) 118:6702–8. 10.1182/blood-2011-08-375006 21979939 PMC3242723

[124] Paczesny S Levine JE Crawford J Choi SW Kitko C Yanik G. Elafin is a biomarker of graft-versus-host disease of the skin. Sci Transl Med. (2010) 2:13ra2. 10.1126/scitranslmed.3000406 20371463 PMC2895410

[125] Dander E Lucchini G Vinci P Introna M Masciocchi F Perseghin P. Mesenchymal stromal cells for the treatment of graft-versus-host disease: understanding the *in vivo* biological effect through patient immune monitoring. Leukemia. (2012) 26:1681–4. 10.1038/leu.2011.384 22289986

[126] von Bahr L Sundberg B Lönnies L Sander B Karbach H Hägglund H. Long-term complications, immunologic effects, and role of passage for outcome in mesenchymal stromal cell therapy. Biol Blood Marrow Transplant. (2012) 18:557–64. 10.1016/j.bbmt.2011.07.023 21820393

[127] Luft T Conzelmann M Benner A Rieger M Hess M Strohhaecker U. Serum cytokeratin-18 fragments as quantitative markers of epithelial apoptosis in liver and intestinal graft-versus-host disease. Blood. (2007) 110:4535–42. 10.1182/blood-2006-10-049817 17702900

[128] Yin F Battiwalla M Ito S Feng X Chinian F Joseph J. Bone marrow mesenchymal stromal cells to treat tissue damage in allogeneic stem cell transplant recipients: Correlation of biological markers with clinical responses. Stem Cells. (2014) 32:1278–88. 10.1002/stem.1638 24452962 PMC3991733

[129] Calkoen FGJ Jol-van der Zijde CM Mearin ML Schweizer JJ Jansen-Hoogendijk AM Roelofs H. Gastrointestinal acute graft-versus-host disease in children: Histology for diagnosis, mesenchymal stromal cells for treatment, and biomarkers for prediction of response. Biol Blood Marrow Transplant. (2013) 19:1590–9. 10.1016/j.bbmt.2013.08.006 23994245

[130] Sánchez-Guijo F Caballero-Velázquez T López-Villar O Redondo A Parody R Martínez C. Sequential third-party mesenchymal stromal cell therapy for refractory acute graft-versus-host disease. Biol Blood Marrow Transplant. (2014) 20:1580–5. 10.1016/j.bbmt.2014.06.015 24952358

[131] von Bonin M Stolzel F Goedecke A Richter K Wuschek N Holig K. Treatment of refractory acute GVHD with third-party MSC expanded in platelet lysate-containing medium. Bone Marrow Transpl. (2009) 43:245–51. 10.1038/bmt.2008.316 18820709

[132] Keto J Kaartinen T Salmenniemi U Castrén J Partanen J Hänninen A. Immunomonitoring of MSC-treated GvHD patients reveals only moderate potential for response prediction but indicates treatment safety. Mol Ther Methods Clin Dev. (2018) 9:109–18. 10.1016/j.omtm.2018.02.001 29516024 PMC5834657

[133] Ankrum JA Ong JF Karp JM. Mesenchymal stem cells: immune evasive, not immune privileged. Nat Biotechnol. (2014) 32:252–60. 10.1038/nbt.2816 24561556 PMC4320647

[134] Lee RH Pulin AA Seo MJ Kota DJ Ylostalo J Larson BL Semprun-Prieto L Delafontaine P Prockop DJ. Intravenous hMSCs Improve Myocardial Infarction in Mice because Cells Embolized in Lung Are Activated to Secrete the Anti-inflammatory Protein TSG-6. Cell Stem Cell. (2009) 5:54–63. 10.1016/j.stem.2009.05.003 19570514 PMC4154377

[135] Meisel R Zibert A Laryea M Gobel U Daubener W Dilloo D. Human bone marrow stromal cells inhibit allogeneic T-cell responses by indoleamine 2,3-dioxygenase-mediated tryptophan degradation. Blood. (2004) 103:4619–21. 10.1182/blood-2003-11-3909 15001472

[136] Su J Chen X Huang Y Li W Li J Cao K. Phylogenetic distinction of iNOS and IDO function in mesenchymal stem cell-mediated immunosuppression in mammalian species. Cell Death Differ. (2014) 21:388–96. 10.1038/cdd.2013.149 24162664 PMC3921585

[137] Choi H Lee RH Bazhanov N Oh JY Prockop DJ. Anti-inflammatory protein TSG-6 secreted by activated MSCs attenuates zymosan-induced mouse peritonitis by decreasing TLR2/NF- B signaling in resident macrophages. Blood. (2011) 118:330–8. 10.1182/blood-2010-12-327353 21551236 PMC3138686

[138] Galleu A Riffo-Vasquez Y Trento C Lomas C Dolcetti L Cheung TS. Apoptosis in mesenchymal stromal cells induces *in vivo* recipient-mediated immunomodulation. Sci Transl Med. (2017) 9:1–12. 10.1126/scitranslmed.aam7828 29141887

[139] Eliopoulos N Stagg J Lejeune L Pommey S Galipeau J. Allogeneic marrow stromal cells are immune rejected by MHC class I- and class II-mismatched recipient mice. Blood. (2005) 106:4057–65. 10.1182/blood-2005-03-1004 16118325

[140] Nauta AJ Westerhuis G Kruisselbrink AB Lurvink EG Willemze R Fibbe WE. Donor-derived mesenchymal stem cells are immunogenic in an allogeneic host and stimulate donor graft rejection in a nonmyeloablative setting. Blood. (2006) 108:2114–20. 10.1182/blood-2005-11-011650 16690970 PMC1895546

[141] Moll G Rasmusson-Duprez I von Bahr L Connolly-Andersen AM Elgue G Funke L. Are therapeutic human mesenchymal stromal cells compatible with human blood? Stem Cells. (2012) 30:1565–74. 10.1002/stem.1111 22522999

[142] Li Y Lin F. Mesenchymal stem cells are injured by complement after their contact with serum. Blood. (2012) 120:3436–43. 10.1182/blood-2012-03-420612 22966167 PMC3482856

[143] Zangi L Margalit R Reich-Zeliger S Bachar-Lustig E Beilhack A Negrin R. Direct imaging of immune rejection and memory induction by allogeneic mesenchymal stromal cells. Stem Cells. (2009) 27:2865–74. 10.1002/stem.217 19750539

[144] Berglund AK Fortier LA Antczak DF Schnabel LV. Immunoprivileged no more: measuring the immunogenicity of allogeneic adult mesenchymal stem cells. Stem Cell Res Ther. (2017) 8:288. 10.1186/s13287-017-0742-8 29273086 PMC5741939

[145] Le Blanc K Rasmusson I Sundberg B Gotherstrom C Hassan M Uzunel M. Treatment of severe acute graft-versus-host disease with third party haploidentical mesenchymal stem cells. Lancet. (2004) 363:1439–41. 10.1016/S0140-6736(04)16104-7 15121408

[146] Ravishankar B Liu H Shinde R Chandler P Baban B Tanaka M. Tolerance to apoptotic cells is regulated by indoleamine 2,3-dioxygenase. Proc Natl Acad Sci USA. (2012) 109:3909–14. 10.1073/pnas.1117736109 22355111 PMC3309765

[147] Mizrahi K Yaniv I Ash S Stein J Askenasy N. Apoptotic signaling through Fas and TNF receptors ameliorates GVHD in mobilized peripheral blood grafts. Bone Marrow Transplant. (2014) 49:640–8. 10.1038/bmt.2014.12 24566711

[148] Morelli AE Larregina AT. Concise review: mechanisms behind apoptotic cell-based therapies against transplant rejection and graft versus host disease. Stem Cells. (2016) 34:1142–50. 10.1002/stem.2326 26865545 PMC4860051

[149] Florek M Sega EI Leveson-Gower DB Baker J Müller AMS Schneidawind D. Autologous apoptotic cells preceding transplantation enhance survival in lethal murine graft-versus-host models. Blood. (2014) 124:1832–42. 10.1182/blood-2014-02-555128 25030062 PMC4162113

[150] Prasad VK Lucas KG Kleiner GI Talano JA Jacobsohn D Broadwater G. Efficacy and safety of *ex vivo* cultured adult human mesenchymal stem cells (Prochymal) in pediatric patients with severe refractory acute graft-versus-host disease in a compassionate use study. Biol Blood Marrow Transpl. (2011) 17:534–41. 10.1016/j.bbmt.2010.04.014 20457269

[151] FrançoisMRomieu-MourezRLiMGalipeauJ. Human MSC suppression correlates with cytokine induction of indoleamine 2,3-dioxygenase and bystander M2 macrophage differentiation. Mol Ther. (2012) 20:187–95. 10.1038/mt.2011.18921934657

[152] Boregowda SV Krishnappa V Haga CL Ortiz LA Phinney DG. A clinical indications prediction scale based on TWIST1 for human mesenchymal stem cells. EBioMedicine. (2016) 4:62–73. 10.1016/j.ebiom.2015.12.020 26981553 PMC4776067

[153] Kuçi Z Bönig H Kreyenberg H Bunos M Jauch A Janssen JWG. Mesenchymal stromal cells from pooled mononuclear cells of multiple bone marrow donors as rescue therapy in pediatric severe steroid-refractory graft-versus-host disease: a multicenter survey. Haematologica. (2016) 101:985–94. 10.3324/haematol.2015.140368 27175026 PMC4967578

[154] Galipeau J Krampera M Barrett J Dazzi F Deans RJ DeBruijn J. International society for cellular therapy perspective on immune functional assays for mesenchymal stromal cells as potency release criterion for advanced phase clinical trials. Cytotherapy. (2016) 18:151–9. 10.1016/j.jcyt.2015.11.008 26724220 PMC4745114

[155] Chinnadurai R Rajan D Qayed M Arafat D Garcia M Liu Y. Potency analysis of mesenchymal stromal cells using a combinatorial assay matrix approach. Cell Rep. (2018) 22:2504–17. 10.1016/j.celrep.2018.02.013 29490284 PMC5855117

[156] Ketterl N Brachtl G Schuh C Bieback K Schallmoser K Reinisch A. A robust potency assay highlights significant donor variation of human mesenchymal stem/progenitor cell immune modulatory capacity and extended radio-resistance. Stem Cell Res Ther. (2015) 6:236. 10.1186/s13287-015-0233-8 26620155 PMC4666276

[157] Perruche S Zhang P Liu Y Saas P Bluestone JA Chen W. CD3-specific antibody–induced immune tolerance involves transforming growth factor-β from phagocytes digesting apoptotic T cells. Nat Med. (2008) 14:528–35. 10.1038/nm1749 18438416

[158] Kleinclauss F Perruche S Masson E de Carvalho Bittencourt M Biichle S Remy-Martin JP. Intravenous apoptotic spleen cell infusion induces a TGF-beta-dependent regulatory T-cell expansion. Cell Death Differ. (2006) 13:41–52. 10.1038/sj.cdd.4401699 15962005 PMC3448559

[159] Fulco T de O Andrade PR Barbosa MG de M Pinto TGT Ferreira PF Ferreira H. Effect of apoptotic cell recognition on macrophage polarization and mycobacterial persistence. Infect Immun. (2014) 82:3968–78. 10.1128/IAI.02194-14 25024361 PMC4187838

[160] Fadok VA Bratton DL Konowal A Freed PW Westcott JY Henson PM. Macrophages that have ingested apoptotic cells *in vitro* inhibit proinflammatory cytokine production through autocrine/paracrine mechanisms involving TGF-beta, PGE2, and PAF. J Clin Invest. (1998) 101:890–8. 10.1172/JCI1112 9466984 PMC508637

[161] Weiss DJ English K Krasnodembskaya A Isaza-Correa JM Hawthorne IJ Mahon BP. The necrobiology of mesenchymal stromal cells affects therapeutic efficacy. Front Immunol. (2019) 10:1228. 10.3389/fimmu.2019.01228 31214185 PMC6557974

[162] Cheung TS Galleu A von Bonin M Bornhäuser M Dazzi F. Apoptotic mesenchymal stromal cells induce prostaglandin E2 in monocytes: implications for the monitoring of mesenchymal stromal cells activity. Haematologica. (2019) 104:e438–441. 10.1097/01.HS9.0000561852.27445.67 30846505 PMC6886441

[163] Valadi H Ekström K Bossios A Sjöstrand M Lee JJ Lötvall JO. Exosome-mediated transfer of mRNAs and microRNAs is a novel mechanism of genetic exchange between cells. Nat Cell Biol. (2007) 9:654–9. 10.1038/ncb1596 17486113

[164] Skog J Würdinger T van Rijn S Meijer DH Gainche L Curry WT. Glioblastoma microvesicles transport RNA and proteins that promote tumour growth and provide diagnostic biomarkers. Nat Cell Biol. (2008) 10:1470–6. 10.1038/ncb1800 19011622 PMC3423894

[165] Théry C Ostrowski M Segura E. Membrane vesicles as conveyors of immune responses. Nat Rev Immunol. (2009) 9:581–93. 10.1038/nri2567 19498381

[166] György B Szabó TG Pásztói M Pál Z Misják P Aradi B. Membrane vesicles, current state-of-the-art: Emerging role of extracellular vesicles. Cell Mol Life Sci. (2011) 68:2667–88. 10.1007/s00018-011-0689-3 21560073 PMC3142546

[167] Stuffers S Sem Wegner C Stenmark H Brech A. Multivesicular endosome biogenesis in the absence of ESCRTs. Traffic. (2009) 10:925–37. 10.1111/j.1600-0854.2009.00920.x 19490536

[168] Colombo M Moita C Van Niel G Kowal J Vigneron J Benaroch P. Analysis of ESCRT functions in exosome biogenesis, composition and secretion highlights the heterogeneity of extracellular vesicles. J Cell Sci. (2013) 126:5553–65. 10.1242/jcs.128868 24105262

[169] Park SJ Kim JM Kim J Hur J Park S Kim K. Molecular mechanisms of biogenesis of apoptotic exosome-like vesicles and their roles as damage-associated molecular patterns. Proc Natl Acad Sci USA. (2018) 115:E11721–30. 10.1073/pnas.1811432115 30463946 PMC6294905

[170] Théry C Witwer KW Aikawa E Alcaraz MJ Anderson JD Andriantsitohaina R. Minimal information for studies of extracellular vesicles 2018 (MISEV2018): a position statement of the International Society for Extracellular Vesicles and update of the MISEV2014 guidelines. J Extracell Vesicles. (2019) 8:1535750. 10.1080/20013078.2018.1535750 30637094 PMC6322352

[171] Gonzalez-Nolasco B Wang M Prunevieille A Benichou G. Emerging role of exosomes in allorecognition and allograft rejection. Curr Opin Organ Transplant. (2018) 23:22–7. 10.1097/MOT.0000000000000489 29189413 PMC5972078

[172] Gunasekaran M Xu Z Nayak DK Sharma M Hachem R Walia R. Donor-derived exosomes with lung self-antigens in human lung allograft rejection. Am J Transplant. (2017) 17:474–84. 10.1111/ajt.13915 27278097 PMC5340154

[173] Zhang H Huang E Kahwaji J Nast CC Li P Mirocha J. Plasma exosomes from HLA-sensitized kidney transplant recipients contain mRNA transcripts which predict development of antibody-mediated rejection. Transplantation. (2017) 101:2419–28. 10.1097/TP.0000000000001834 28557957

[174] Tower CM Reyes M Nelson K Leca N Kieran N Muczynski K. Plasma C4d+ endothelial microvesicles increase in acute antibody-mediated rejection. Transplantation. (2017) 101:2235–43. 10.1097/TP.0000000000001572 27846156

[175] Alvarez S Suazo C Boltansky A Ursu M Carvajal D Innocenti G. Urinary exosomes as a source of kidney dysfunction biomarker in renal transplantation. Transplant Proc. (2013) 45:3719–23. 10.1016/j.transproceed.2013.08.079 24315007

[176] Sigdel TK Ng YW Lee S Nicora CD Qian WJ Smith RD. Perturbations in the urinary exosome in transplant rejection. Front Med. (2015) 2:1–10. 10.3389/fmed.2014.00057 25593928 PMC4292055

[177] Gregson AL Hoji A Injean P Poynter ST Briones C Palchevskiy V. Altered exosomal RNA profiles in bronchoalveolar lavage from lung transplants with acute rejection. Am J Respir Crit Care Med. (2015) 192:1490–503. 10.1164/rccm.201503-0558OC 26308930 PMC4731719

[178] Collino F Deregibus MC Bruno S Sterpone L Aghemo G Viltono L. Microvesicles derived from adult human bone marrow and tissue specific mesenchymal stem cells shuttle selected pattern of miRNAs. PLoS ONE. (2010) 5:e11803. 10.1371/journal.pone.0011803 20668554 PMC2910725

[179] Kim J Breunig MJ Escalante LE Bhatia N Denu RA Dollar BA. Biologic and immunomodulatory properties of mesenchymal stromal cells derived from human pancreatic islets. Cytotherapy. (2012) 14:925–35. 10.3109/14653249.2012.684376 22571381 PMC3537170

[180] Harting MT Srivastava AK Zhaorigetu S Bair H Prabhakara KS Toledano Furman NE Inflammation-stimulated mesenchymal stromal cell-derived extracellular vesicles attenuate inflammation. Stem Cells. (2017) 6:79–90. 10.1002/stem.2730 29076623

[181] Ruppert KA Nguyen TT Prabhakara KS Toledano Furman NE Srivastava AK Harting MT. Human mesenchymal stromal cell-derived extracellular vesicles modify microglial response and improve clinical outcomes in experimental spinal cord injury. Sci Rep. (2018) 8:480. 10.1038/s41598-017-18867-w 29323194 PMC5764957

[182] Di Trapani M Bassi G Midolo M Gatti A Kamga PT Cassaro A. Differential and transferable modulatory effects of mesenchymal stromal cell-derived extracellular vesicles on T, B and NK cell functions. Sci Rep. (2016) 6:24120. 10.1038/srep24120 27071676 PMC4829861

[183] Showalter MR Wancewicz B Fiehn O Archard JA Clayton S Wagner J. Primed mesenchymal stem cells package exosomes with metabolites associated with immunomodulation. Biochem Biophys Res Commun. (2019) 512:729–35. 10.1016/j.bbrc.2019.03.119 30926165 PMC6682414

[184] Lynch C Panagopoulou M Gregory CD. Extracellular vesicles arising from apoptotic cells in tumors: roles in cancer pathogenesis and potential clinical applications. Front Immunol. (2017) 8:1174. 10.3389/fimmu.2017.01174 29018443 PMC5614926

[185] Caruso S Poon IKH. Apoptotic cell-derived extracellular vesicles: more than just debris. Front Immunol. (2018) 9:1486. 10.3389/fimmu.2018.01486 30002658 PMC6031707

[186] Lalu MM McIntyre L Pugliese C Fergusson D Winston BW Marshall JC Safety of cell therapy with mesenchymal stromal cells (SafeCell): a systematic review and meta-analysis of clinical trials. PLoS ONE. (2012) 7:e47559 10.1371/journal.pone.0047559 23133515 PMC3485008

[187] Trento C Bernardo ME Nagler A Kuçi S Bornhäuser M Köhl U. Manufacturing mesenchymal stromal cells for the treatment of graft-versus-host disease: a survey amongst centers affiliated to the European group of blood and marrow transplantation. Biol Blood Marrow Transplant. (2018) 24:2365–70. 10.1016/j.bbmt.2018.07.015 30031938 PMC6299357

